# Case report long segment myelitis secondary to neuro melioidosis

**DOI:** 10.1186/s12883-022-02917-6

**Published:** 2022-10-19

**Authors:** Sahathevan Vithoosan, Asha Kumarasiri, Nadun Madushanka Vithanage, Bimsara Senanayake

**Affiliations:** Senior Registrar in Neurology, National Hospital, No. 15B1, Campbell Place, Dehiwela, Sri Lanka Colombo Sri Lanka

**Keywords:** Long segment myelitis, Melioidosis, *Burkholderia pseudomallei*

## Abstract

**Background:**

Neuro-melioidosis, comprising 4% of all cases of melioidosis carries a risk of high morbidity and mortality. We describe two Sri Lankan patients presenting with long segment myelitis secondary to melioidosis.

**Case presentation:**

Case 1: 47-year-old male presented with right side hemiparesis which progressed rapidly to quadriparesis. Initial cerebro spinal fluid (CSF) analysis revealed protein 76 mg/dl and glucose 72 mg/dl but without a cellular reaction. MRI spine revealed long segment myelitis with contrast enhancement. The patient was treated with intravenous methyl prednisolone pulses (IV MPP) and plasma exchanges(PLEX) on suspicion of an immune mediated myelitis but without success. A repeat MRI revealed high signal changes in the brain stem and along the entire spinal cord with contrast enhancement. MRI brain after treatment with MPP/PLEX showed enhancing hyper intensities along the corticospinal tracts. The repeat CSF revealed protein 1187 mg/dl, glucose 78 mg/dl, lymphocytes 1600/mm3 and neutrophils 10,200/mm3. CSF culture has become positive for *Burkholderia pseudomallei*. Serum melioidosis antibody titre was 1: 320. He was started on IV meropenem with oral cotrimoxazole for 12 weeks followed by oral co trimoxazole. But he had poor clinical recovery.

Case 2: 47-year-old female presented with bilateral lower limb weakness for 1-week duration. On examination, she had flaccid paraparesis with a sensory level at T11. Inflammatory markers were elevated. CSF analysis revealed protein 50 mg/dl with lymphocytes 172/mm3. MRI pan spine revealed a long segment myelitis. Serum melioidosis antibody titre was 1: 640. She was treated with IV meropenem for 8 weeks followed by oral co-trimoxazole with an excellent clinical and radiological response.

**Conclusion:**

Numerous neurological manifestations have been described with melioidosis, however long segment myelitis with a positive CSF culture is not yet reported. These cases signify the importance of considering melioidosis as a differential in patients with long segment myelitis especially in endemic areas.

## Background

Melioidosis is an emerging infection in South East Asia and Australia [1]. It has a wide range of clinical presentations, especially in immunocompromised patients [2]. Melioidosis is contracted by inoculation of soil and water through wounds or inhalation. It was first described in 1912 by Whitmore and Krishnaswami [3, 4] in Myanmar. Afterwards, Sri Lanka was one of the first countries to report melioidosis in 1927 [5]. Melioidosis involves the pulmonary and genitourinary systems, along with bone and soft tissue, sometimes causing severe sepsis. Neurologic complications (neuro-melioidosis) are seen in approximately 4% of all cases, making it clinically important to diagnose early because of its high mortality rate of approximately 25% and significant morbidity [6]. Neuro-melioidosis has numerous presentations. It can mimic Guillain–Barre syndrome and present with limb weakness and cranial nerve palsies. Further, it can have features of meningoencephalitis, with fever, headache, neck stiffness, altered consciousness and seizures. The facial nerve is the most common cranial nerve affected. Most central nervous system (CNS) melioidosis patients (91%) were classified as acute melioidosis (less than two months of onset). Even though various presentations of neuro-melioidosis have been described, long segment myelitis due to melioidosis has not been reported so far. Here, we describe two cases of long segment myelitis secondary to melioidosis and highlight the importance of considering this differential when evaluating such cases.

## Case presentation

### Case 1

**Fig. 1:**
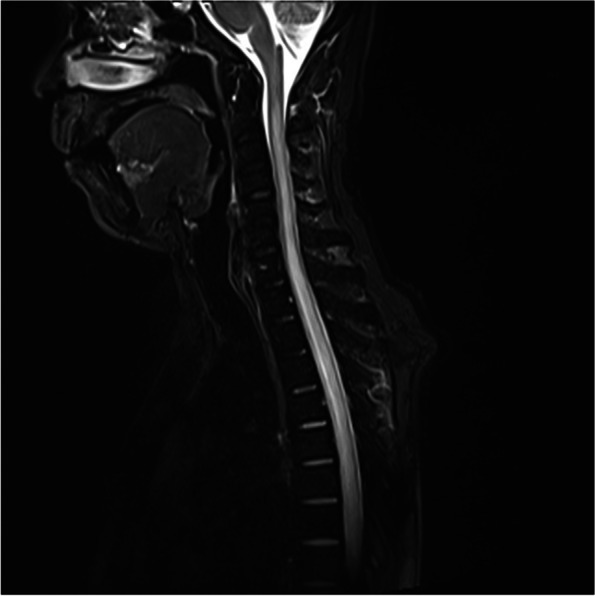
T2W fat supressed MRI spine shows T2 high signal extending from cervico medullary junction to the thoracic spinal cord

**Fig. 2:**
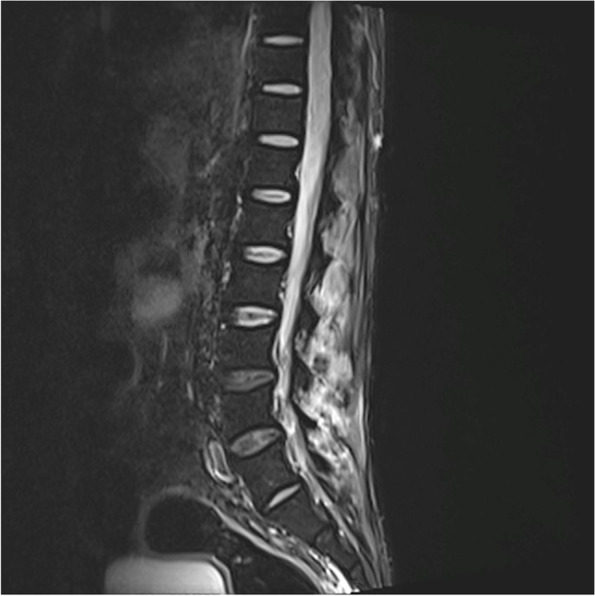
T2W fat suppressed MRI spine shows continuation of the T2 high signal up to the conus medullaris

**Fig. 3:**
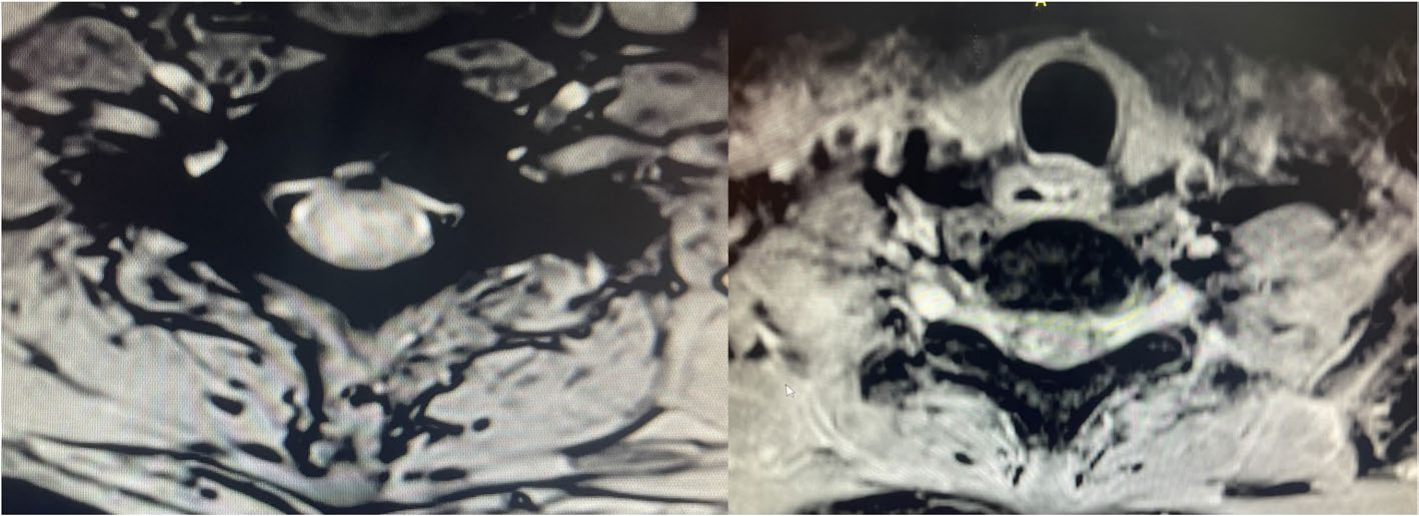
T2W Fat suppressed MRI spine axial image at C6 level showing central hyperintensity and post contrast image showing contrast enhancement

**Fig. 4:**
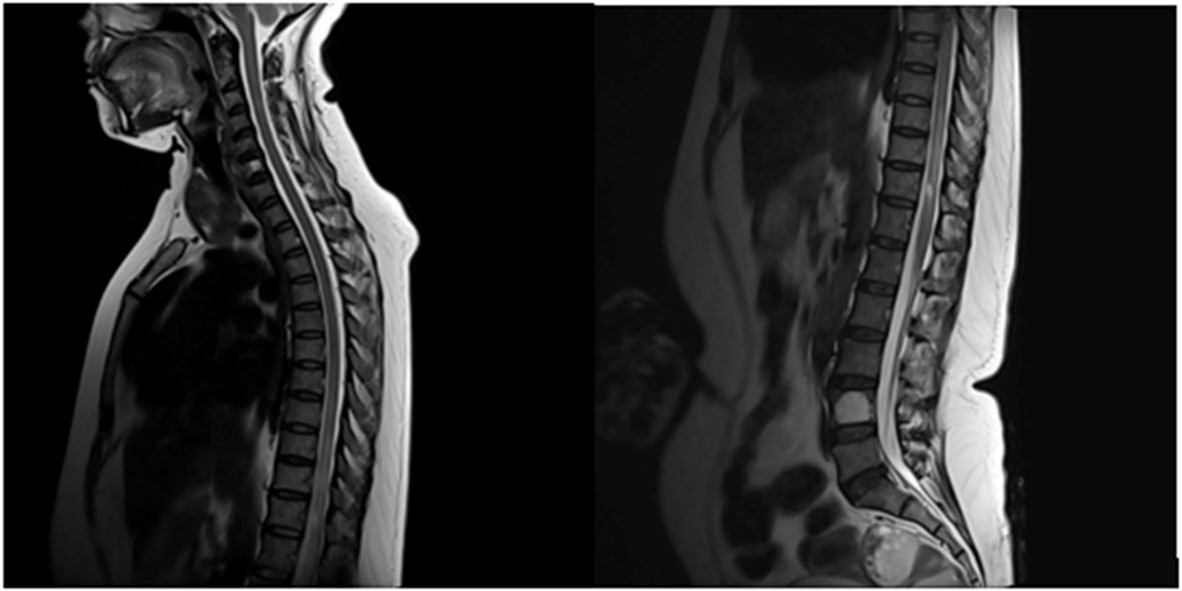
T1 fat saturated contrast MRI spine shows contrast enhancement of the whole spinal cord

**Fig. 5:**
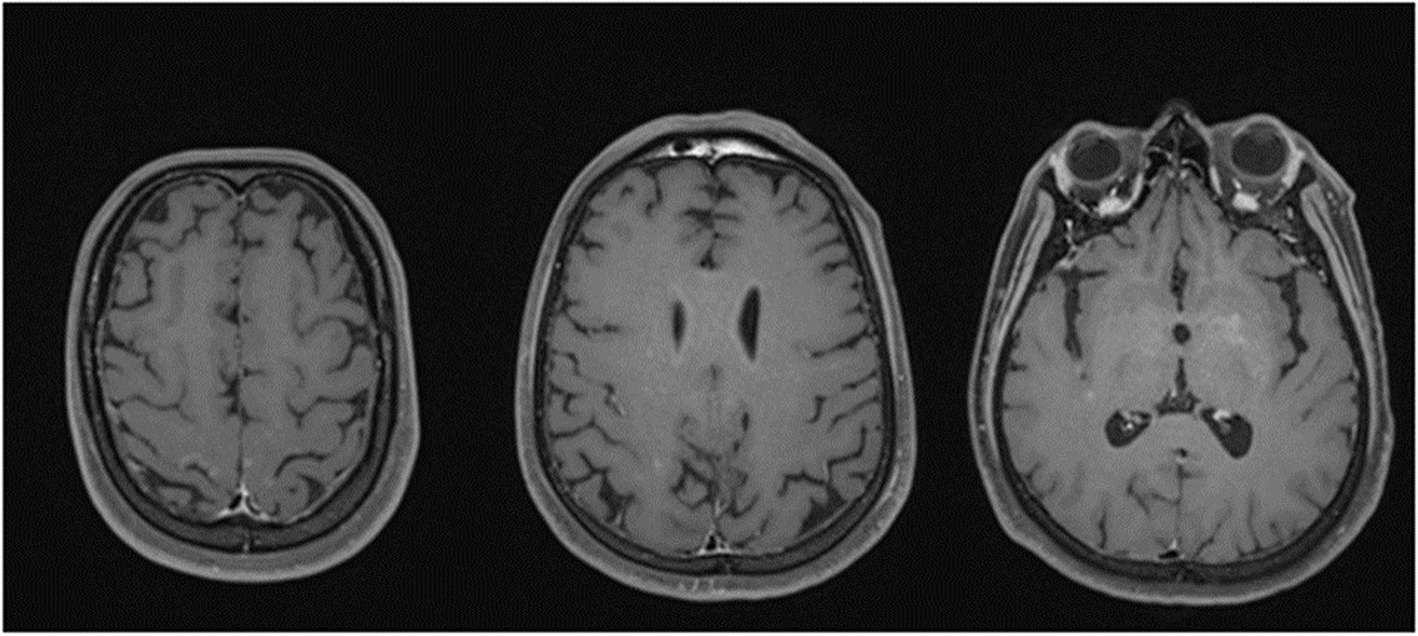
T1 contrast axial image of MRI brain shows linear enhancement along the corticospinal tracts

**Fig. 6:**
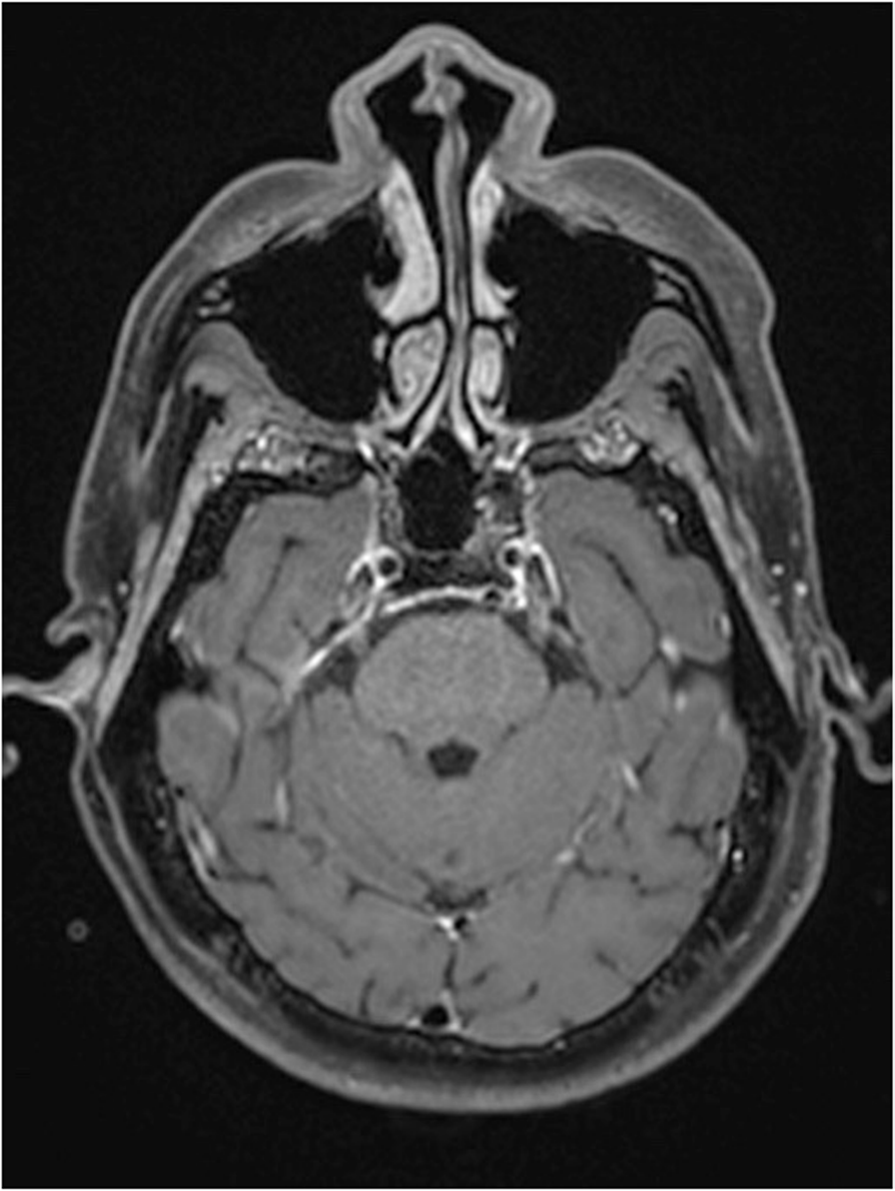
Contrast MRI brain shows mild contrast enhancement of trigeminal nerves bilaterally

A 47-year-old post man presented with right-sided upper and lower limb weakness, which rapidly progressed to quadriparesis with bladder and bowel involvement. He had no history of fever but had recurrent hiccups. There was no preceding history of tuberculosis. The patient had no history of recent vaccination. There was no significant local or international travel history. An examination revealed flaccid quadriparesis. The upper limb power was MRC grade 3/5 bilaterally and the bilateral lower limb power was MRC grade 0/5. Reflexes were diminished in the upper and lower limbs and he had bilateral up going plantar response. There was no obvious sensory level. Rest of the neurological examination including cranial nerves was normal. The initial haematology screen including inflammatory markers was unremarkable. Repeated blood cultures were sterile. Initial CSF analysis revealed protein 76 mg/dl and 72 mg glucose/dl (Random Blood Glucose 159 mg/dl) but without a cellular reaction. CSF Tuberculosis Polymerase Chain Reaction (TB PCR)/gene Xpert was negative. CSF oligo clonal bands were negative. The initial CSF bacterial culture did not reveal any organisms. A spine MRI showed an enhacing long segment myelitis extending from the lower medulla up to the T10 level (Figs. 1, 2 and 3). The brain MRI was initially normal. Neuromyelitis Optica Spectrum Disorder (NMOSD) with AQP4-IgG was considered the most likely possibility; hence, he was treated with IV methyl prednisolone pulses 1 g daiily for 5 days followed by plasma exchange (PLEX), but without success. However,serum NMO and MOG antibodies were found to be negative later and the visual evoked potential (VEP) test was normal excluding subclinical optic neuritis. A repeat MRI revealed an abnormally high signal in the brain stem and along the entire spinal cord with contrast enhancement (Fig. 4). The brain MRI thereafter showed linear enhancement along the corticospinal tracts (Fig. 5). Trigeminal nerves bilaterally showed mild contrast enhancement (Fig. 6). The repeat CSF study revealed protein 1,187 mg/dl, glucose 78 mg/dl (RBS 198), lymphocytes 1,600/mm^3^ and neutrophils 10,200/ mm^3^. CSF-Adenosine De Aminase (ADA) was elevated 26 U/L. With the significant CSF glucose drop, cellular reaction and elevated CSF ADA, CNS-TB was considered, and anti-TB treatment was started. However, subsequently, the CSF culture became positive for *Burkholderia pseudomallei*. The serum melioidosis antibody titre was 1: 320. IV meropenem was initiated with oral cotrimoxazole for 12 weeks followed by maintenance with oral cotrimoxazole. Regardless, the clinical recovery was poor, with persistent quadriparesis despite some imaging improvements.

### Case 2

**Fig. 7:**
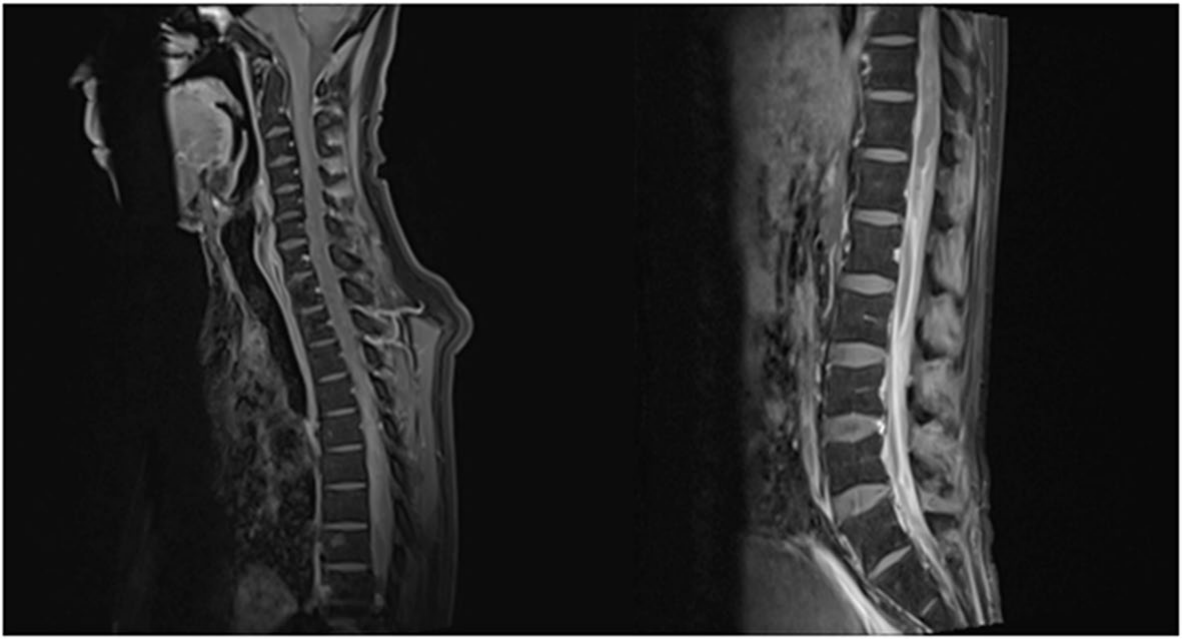
T2W sagittal image of the MRI pan spine revealed patchy central long segment T2 hyper intensity in the thoracic spinal cord extending from T6 level to conus medullaris

**Fig. 8:**
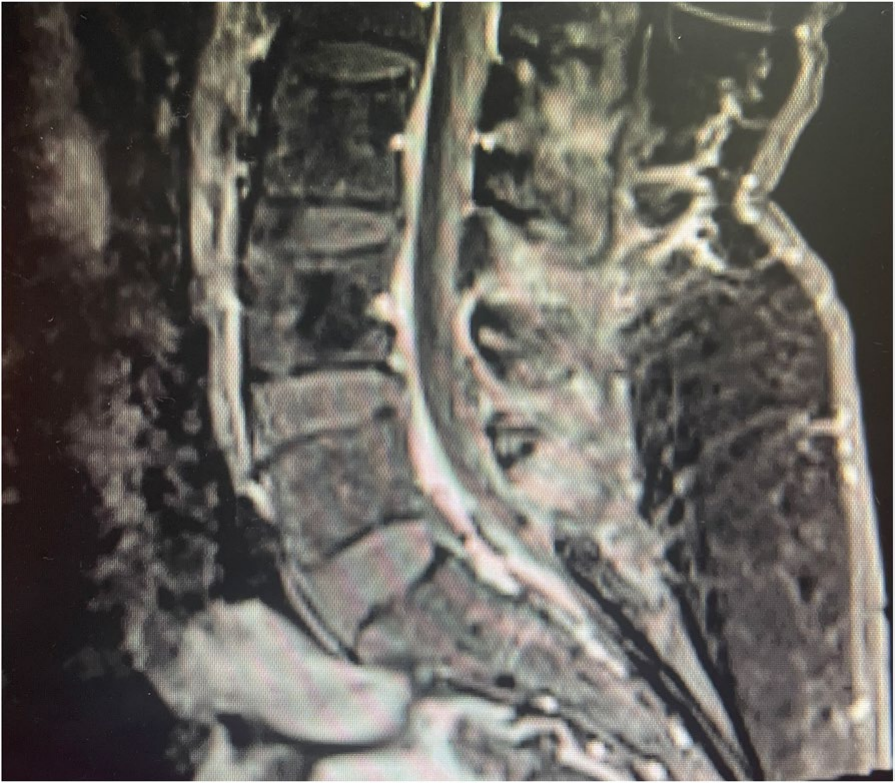
Sagittal image of the MRI spine revealing contrast enhancement of the cauda equina and anterior dura

A 47-year-old housewife presented with bilateral lower limb weakness of 1 week. She had no history of recent infections, vaccination or travel. On examination, bilateral flaccid paraparesis with a sensory level at the T11 level was noted. The lower limb power was MRC grade 4/5 bilaterally with diminished reflexes. However, she had bilateral up going plantar response. Upper limb and cranial nerves neurological examinations were normal. Inflammatory markers were elevated. The blood culture was sterile. CSF analysis revealed protein 50 mg/dl with lymphocytes 172/mm^3^ without a glucose drop and a normal ADA. CSF oligo clonal bands were negative. A pan spine MRI revealed a patchy central long segment T2 hyper intensity in the thoracic spinal cord extending from the T6 level to the conus medullaris (Fig. 7). Lesions were located in the ventral and right lateral region of the thoracic spinal cord (Fig. 7). Furthermore, contrast enhancement of the cauda equina was demonstrated, in addition to anterior dural enhancement (Fig. 8). The serum melioidosis antibody titre was 1: 640. Serum NMO and MOG antibodies were found to be negative later and the visual evoked potential test was normal. The patient was treated with IV meropenem for 8 weeks followed by oral cotrimoxazole 1,920 mg bd. The patient had an excellent clinical response with a near-complete clinical recovery and resolution of MRI changes.

## Discussion and conclusions

Longitudinally extensive transverse myelitis (LETM) has many differentials, with AQP4-NMOSD being the most common. Other possible aetiologies include Systemic Lupus Erythematous (SLE), Sjogren’s syndrome, vitamin B12 deficiency, intrathecal tumours and such vascular abnormalities as AVM, infective causes like HIV 1 and HTLV and, rarely, intramedullary TB. LETM due to melioidosis has not been reported so far to the best of our knowledge.

Melioidosis, which is caused by gram-negative, facultative, soil saprophyte *Burkholderia pseudomallei*, is contracted by inoculation of soil and water through wounds or inhalation and is particularly common during and after the wet season. Risk factors for melioidosis are widely documented and include diabetes mellitus, alcoholism, renal disease, immunosuppression and thalassemia [7]. This is most likely due to compromised cell-mediated immunity in such patients. However, these risk factors can be absent in patients with neuro-melioidosis [8], as in our cases. A history of exposure to infected soil was absent; in our first case, however, the second patient had worked in the paddy field three months prior to the onset of symptoms. *B*. *pseudomallei* infection can be latent for extended periods and then become reactivated, like CNS tuberculosis. Melioidosis is also known as the ‘Vietnamese time bomb’, as it has been reported in US soldiers who served in Vietnam several years before the onset of meningitis due to CNS melioidosis [9].

In neuro-melioidosis, CSF findings usually show mononuclear cells, high CSF protein and normal glucose levels [8]. In our first case, CSF was initially acellular but later became predominantly neutrophilic. An elevated protein level and a significant glucose drop were also noted. The CSF ADA too was elevated (26U/L). This prompted initial anti-TB therapy, which was revised after the *B*. *pseudomallei*-positive CSF culture report. In the second case, a lymphocytic CSF with elevated protein without a sugar drop was consistent with the commonly found changes in CNS melioidosis. Hence, CSF findings in CNS-Melioidosis can vary. A very high CSF- ADA level (> 10U/L) is of some value in the diagnosis of TB Meningitis (TBM) [10]. However, CSF- ADA levels in CNS melioidosis must be further studied before considering its value as part of the diagnostic work up.

The long segment myelitis seen in the spinal cord of both these patients was attributed to neuro- melioidosis, having excluded all other causes. Positive CSF culture and elevated melioidosis antibodies supported the diagnosis. CNS melioidosis shows a propensity to involve and spread along the white matter tracts across the commissural or longitudinal fibres [8]. The spread of micro abscesses along white matter tracts and frequent trigeminal nerve involvement are unique imaging characteristics of CNS melioidosis [11]. These findings were noted in the repeat MRI of our first patient.

Therefore, the diagnosis of CNS melioidosis depends on various factors. First, the risk factors must be considered even though they can be absent. CSF findings can vary and can be nonspecific, as described in our patients (Table 1). However, elevated protein without a significant glucose drop with mononuclear cells may suggest a CNS melioidosis diagnosis. A rising titre of melioidosis antibodies too will point to the diagnosis. White matter tract involvement in brain MRI is another useful tool [8].

**Table 1:** Comparison of the clinical features, investigation findings and the treatment response of both patients

	1^st^ Patient	2^nd^ Patient
Clinical Presentation	Right sided upper and lower limb weakness rapidly evolving into quadripareseis with brainstem involvement	Bilateral lower limb paraparesis
CSF Findings	Protein – Elevated CSF Glucose drop (< 50% of blood glucose)- present Cells- No cellular reaction in 1^st^ sample, Significantly increased neutrophils in 2^nd^ sample CSF ADA- Elevated 26U/L	Protein – Elevated CSF Glucose drop(< 50% of blood glucose)- Absent Cells- Elevated lymphocytes CSF ADA- Normal
MRI Spine	Long segment myelitis with contrast enhancement extending up to medulla	Long segment myelitis with contrast enhancement
MRI Brain	1^st^ Report- Normal 2^nd^ Report- linear enhancement along the corticospinal tracts, Contrast enhancement of trigeminal nerves	Normal
CSF Culture	positive for *Burkholderia pseudomallei*	Negative for *Burkholderia pseudomallei*
Serum melioidosis antibody	1:320	1:640
Treatment response	Poor clinical response	Excellent clinical response

Delayed diagnosis and extensive involvement of the spinal cord and brain can make the prognosis poor in this condition, as in our first case (Table 1). On the other hand, an early diagnosis with the initiation of appropriate therapy may result in a favourable outcome. Hence, we propose that neuro-melioidosis be considered an important differential when evaluating long segment myelitis, especially in endemic areas.

## Data Availability

The datasets supporting the conclusions of this article are included in the article.

## Abbreviations

MRI: Magnetic Resonance Imaging
CSF: Cerebro spinal fluid
RBS: Random blood sugar
IV MPP: Intravenous methyl prednisolone pulses
PLEX: Plasma exchange
ESR: Erythrocyte Sedimentation Rate
CRP: C Reactive Protein
ADA: Adenosine De Aminase
CECT: Contrast Enhanced Computerised Tomography

## References

[1] Gibney KB, Cheng AC, Currie BJ (2008). Cutaneous Melioidosis in the Tropical Top End of Australia: A Prospective Study and Review of the Literature. Clin Infect Dis.

[2] Perera GND, Dias LD, Kulatunga A, Corea E, Masakorala J (2012). A case report of melioidosis. Sri Lankan J Infect Dis.

[3] ‌Cheng, A. C., and B. J. Currie.  (2005). Melioidosis: epidemiology, pathophysiology, and management. Clin Microbiol Rev.

[4] White NJ (2003). Melioidosis. Lancet (London, England).

[5] Thin R.N, Brown M, Stewart J.B, Garrett C. J (1970). “Melioidosis: A Report of Ten Cases.”. The Quarterly Journal of Medicine.

[6] Chadwick DR, Ang B, Sitoh YY, Lee CC (2002). Cerebral melioidosis in Singapore: a review of five cases. Trans R Soc Trop Med Hyg.

[7] Suputtamongkol Y, Chaowagul W, Chetchotisakd P, Lertpatanasuwun N, Intaranongpai S, Ruchutrakool T, Budhsarawong D (1999). Risk factors for melioidosis and bacteremic melioidosis. Clin Infect Dis.

[8] Currie BJ, Fisher DA, Howard DM, James NC, Burrow (2000). Neurological melioidosis. Acta Trop.

[9] Beck RW, Janssen RS, Smiley ML, Schatz NJ, Savino PJ, Rubin DH (1984). Melioidosis and bilateral third-nerve palsies. Neurology.

[10] Gupta B (2010). Adenosine deaminase levels in CSF of tuberculous meningitis patients. J Clin Med Res.

[11] Hsu  Charlie Chia-Tsong,  Singh Dalveer, Kwan Gigi, Deuble Martin, Aquilina Chloe, Korah Ipeson, Norton Robert (2015). “Neuromelioidosis: Craniospinal MRI Findings InBurkholderia PseudomalleiInfection.”. J Neuroimaging.

